# Supplementary material for: A Biocompatible Liquid Pillar[n]arene-Based Drug Reservoir for Topical Drug Delivery
Source: Pharmaceutics. 2022 Nov 28;14(12):2621. doi: 10.3390/pharmaceutics14122621 (PMC9783689; doi:10.3390/pharmaceutics14122621)
Supplement: Supplementary file 1 [file pharmaceutics-14-02621-s001.zip › pharmaceutics-2026812-supplementary.pdf]

Supporting Information

Biocompatible Liquid Pillar[n]arene-Based Drug Reservoir for Topical Administration

Yahan Zhang [1], Mengke Ma [1], Longming Chen [1], Xinbei Du [1], Zhao Meng [1], Han Zhang [1], Zhibing Zheng [1,*], Junyi Chen [1,2,*] and Qingbin Meng [1,*]

[1] State Key Laboratory of Toxicology and Medical Countermeasures, Beijing Institute of Pharmacology and Toxicology, Beijing 100850, China

[2] Key Laboratory of Inorganic-Organic Hybrid Functional Material Chemistry, Ministry of Education, Tianjin Key Laboratory of Structure and Performance for Functional Molecules, College of Chemistry, Tianjin Normal University, Tianjin 300387, China

Table of Contents

1. Synthesis of OPEns S2

2. Supporting results and experimental raw data S5

2.1 ^{1}H NMR, ^{13}C NMR and MALDI-TOF-MS spectra of OEPns S5

2.2 Viscoelastic spectra for OEPns S14

2.3 H&E analyses of skin tissue S15

2.4 Optimized geometries of ECN with TEP6 S16

2.5 Calibration curves of ECN S17

3. References S18

1. Synthesis of pillar[n]arene with oligoethylene oxide substituents.

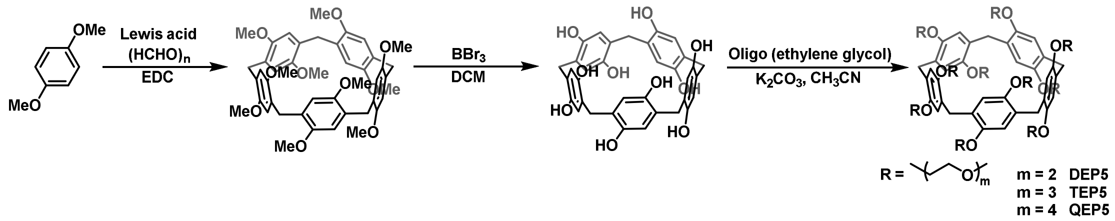

Scheme S1. Synthesis of pillar[5]arene modified with oligoethylene oxide substituents (OEP5).

Synthesis of *per*-hydroxylated pillar[5]arene: *per*-Hydroxylated pillar[5]arene were synthesized according to the previous paper.[1]

Synthesis of OEP5: In brief, DEP5 for example, to a solution of *per*-hydroxylated pillar[5]arene (0.58 g, 1 mmol) in acetone (30 mL), potassium carbonate (4.14 g, 30 mmol) was added. Then, diethylene glycol monomethyl ether mono-*p*-tosylate (5.48 g, 20 mmol) was added and heated at 60 ^{o}C for 3 d. After filtration, the solvent was removed by evaporation. The residue was purified by column chromatography on silica gel to afford DEP5 (0.64 g, 39%). TEP5 (0.77 g, 35%) and QEP5 (0.80g, 32%) were afforded according to the same method.

DEP5: ^{1}H NMR ($CDCl_3$, 600 MHz, ppm): δ 6.83 (s, 10H), 4.01 (t, 20H), 3.81 (t, 20H), 3.76 (s, 10H), 3.70 (t, 20H), 3.51 (t, 20H), 3.27 (s, 30H); ^{13}C NMR ($CDCl_3$, 150 MHz, ppm): δ 148.77, 127.80, 114.32, 71.04, 69.62, 69.17, 67.09, 57.92, 28.34; HRMS (MALDI-TOF-MS) (m/z): $[M+Na]^+$ calcd. for $C_{85}H_{130}O_{30}$, 1653.94, found 1653.614.

TEP5: ^{1}H NMR ($CDCl_3$, 600 MHz, ppm): δ 6.83 (s, 10H), 4.01 (t, 20H), 3.83 (s, 20H), 3.73 (m, 24H), 3.65 (t, 20H), 3.59 (t, 20H), 3.45 (t, 30H), 3.28 (s, 30H); ^{13}C NMR ($CDCl_3$, 150 MHz, ppm): δ 149.96, 128.91, 115.42, 70.95, 70.90, 70.72, 70.42, 68.19, 59.13, 29.45; HRMS (MALDI-TOF-MS) (m/z): $[M+Na]^+$ calcd. for $C_{105}H_{170}O_{40}$, 2093.47, found 2093.63.

QEP5: ^{1}H NMR ($CDCl_3$, 600 MHz, ppm): δ 6.82 (s, 10H), 4.00 (t, 20H), 3.82 (t, 20H), 3.73 (m, 30H), 3.65 (t, 20H), 3.61 (t, 20H), 3.58 (m, 40H), 3.49 (t, 20H), 3.32 (s, 30H). ^{13}C NMR ($CDCl_3$, 150 MHz, ppm): δ 148.80, 127.69,114.27, 70.88, 69.73, 69.61, 69.53, 69.48, 69.44, 69.20, 67.03, 57.03, 57.96, 28.69; HRMS (MALDI-TOF-MS) (m/z): $[M+Na]^+$ calcd. for $C_{125}H_{210}O_{50}$, 2535.46, found 2534.03.

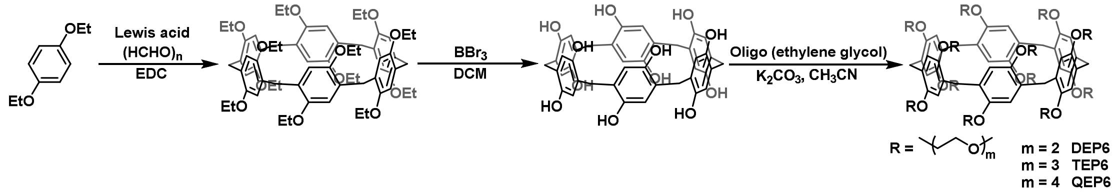

Synthesis of *per*-hydroxylated pillar[6]arene: *per*-hydroxylated pillar[6]arene were obtained according to the same method of *per*-hydroxylated pillar[5]arene.

Synthesis of OEP6: In brief, DEP6 for example, to a solution of *per*-hydroxylated pillar[6]arene (0.70 g, 1 mmol) in acetone (30 mL), potassium carbonate (4.82 g, 36 mmol) was added. Then, diethylene glycol monomethyl ether mono-*p*-tosylate (6.58 g, 24 mmol) was added and heated at 60 °C for 3 d. After filtration, the solvent was removed by evaporation. The residue was purified by column chromatography on silica gel to afford DEP6 (0.59 g, 30%). The method of TEP6 (0.70 g, 28%) and QEP6 (0.75 g, 25%) was the same as that

of DEP6.

DEP6: ^{1}H NMR ($CDCl_3$, 600 MHz, ppm): δ 6.70 (s, 12H), 3.93 (t, 24H), 3.78 (s, 12H), 3.72 (t, 24H), 3.65 (t, 24H), 3.52 (t, 24H), 3.35 (s, 36H); ^{13}C NMR ($CDCl_3$, 150 MHz, ppm): δ 149.53, 127.18, 114.55, 70.97, 69.58, 69.02, 67.37, 57.98, 29.94; HRMS (MALDI-TOF-MS) (m/z): $[M+Na]^+$ calcd. for $C_{102}H_{156}O_{36}$, 1980.32, found 1979.57

TEP6: ^{1}H NMR ($CDCl_3$, 600 MHz, ppm): δ 6.69 (s, 12H), 3.91 (t, 24H), 3.77 (s, 12H), 3.67 (m, 48H), 3.63 (t, 48H), 3.52 (t, 24H), 3.34 (s, 36H). ^{13}C NMR ($CDCl_3$, 150 MHz, ppm): δ 150.56, 128.20, 115.63, 71.91, 70.68, 70.62, 70.51, 70.02, 68.38, 58.99, 30.99; HRMS (MALDI-TOF-MS) (m/z): $[M+Na]^+$ calcd. for $C_{126}H_{204}O_{48}$, 2508.96, found 2507.97.

QEP6: ^{1}H NMR ($CDCl_3$, 600 MHz, ppm): δ 6.69 (s, 12H), 3.91 (t, 24H), 3.76 (s, 12H), 3.70 (t, 24H), 3.66 (t, 24H), 3.65 (t, 24H), 3.60 (m, 96H), 3.60 (t, 24H), 3.52 (s, 36H); ^{13}C NMR ($CDCl_3$, 150 MHz, ppm): δ 149.52, 127.09, 114.53, 70.90, 69.67, 69.69, 68.58, 69.57, 69.49, 69.01, 67.31, 58.00, 30.01; HRMS (MALDI-TOF-MS) (m/z): $[M+Na]^+$ calcd. for $C_{150}H_{252}O_{60}$, 3036.60 found 3035.90.

2. Supporting results and experimental raw data

2.1 ^{1}H NMR, ^{13}C NMR and MALDI-TOF-MS spectra of OEPns

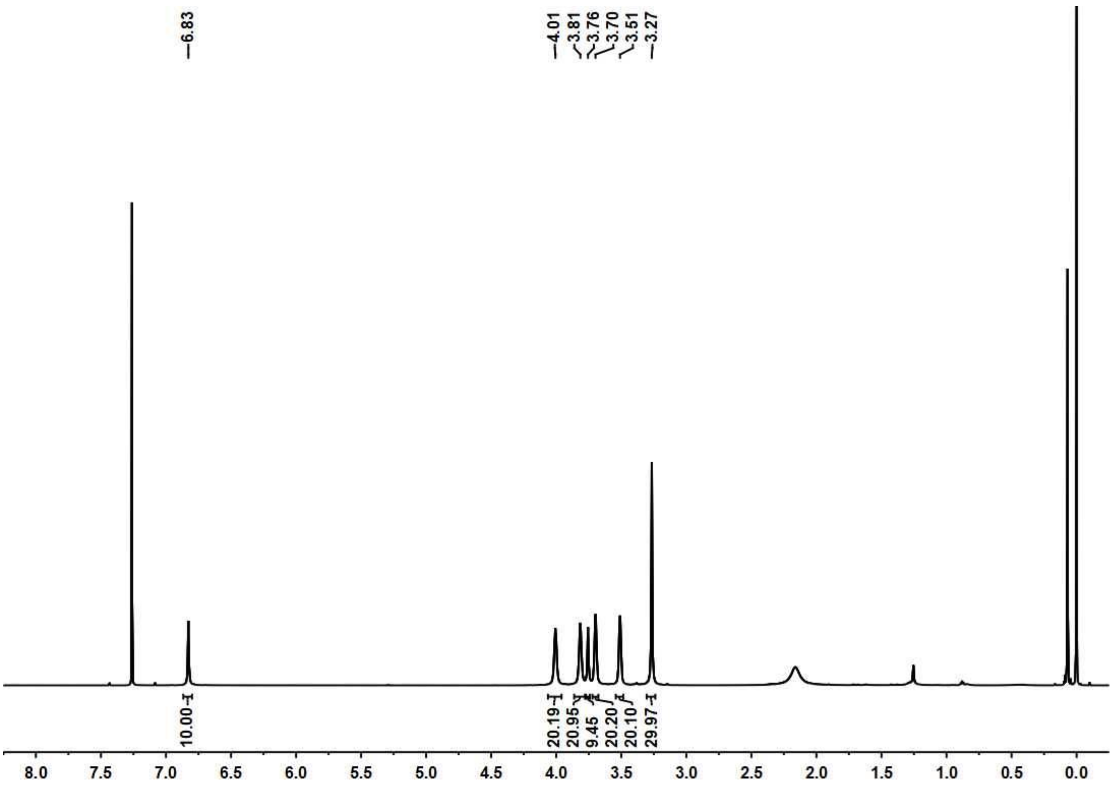

Figure S1. ^{1}H NMR spectrum (600 MHz, $CDCl_3$) of DEP5.

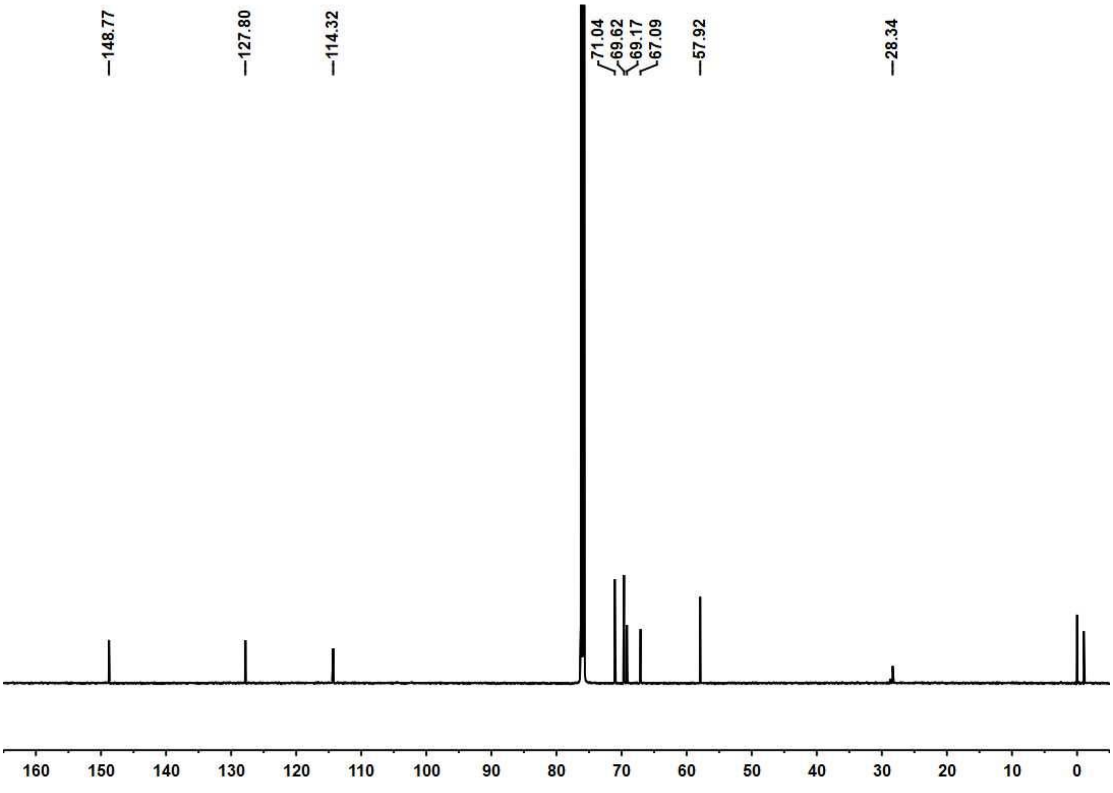

Figure S2. ^{13}C NMR spectrum (150 MHz, $CDCl_3$) of DEP5.

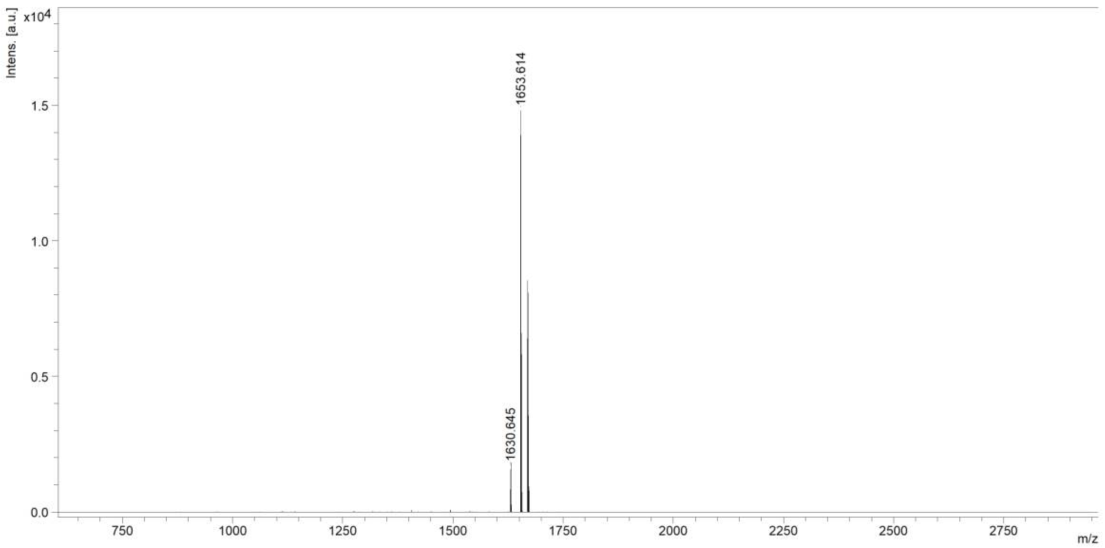

Figure S3. MALDI-TOF-MS of DEP5.

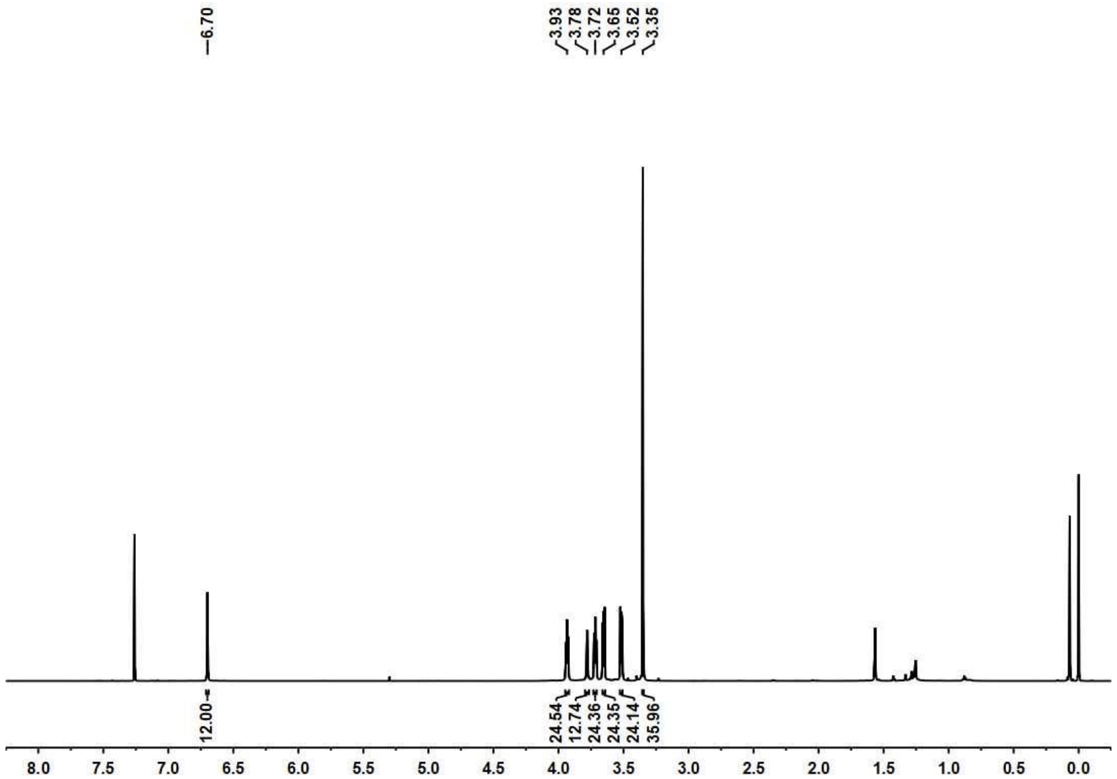

Figure S4. ^{1}H NMR spectrum (600 MHz, $CDCl_3$) of DEP6.

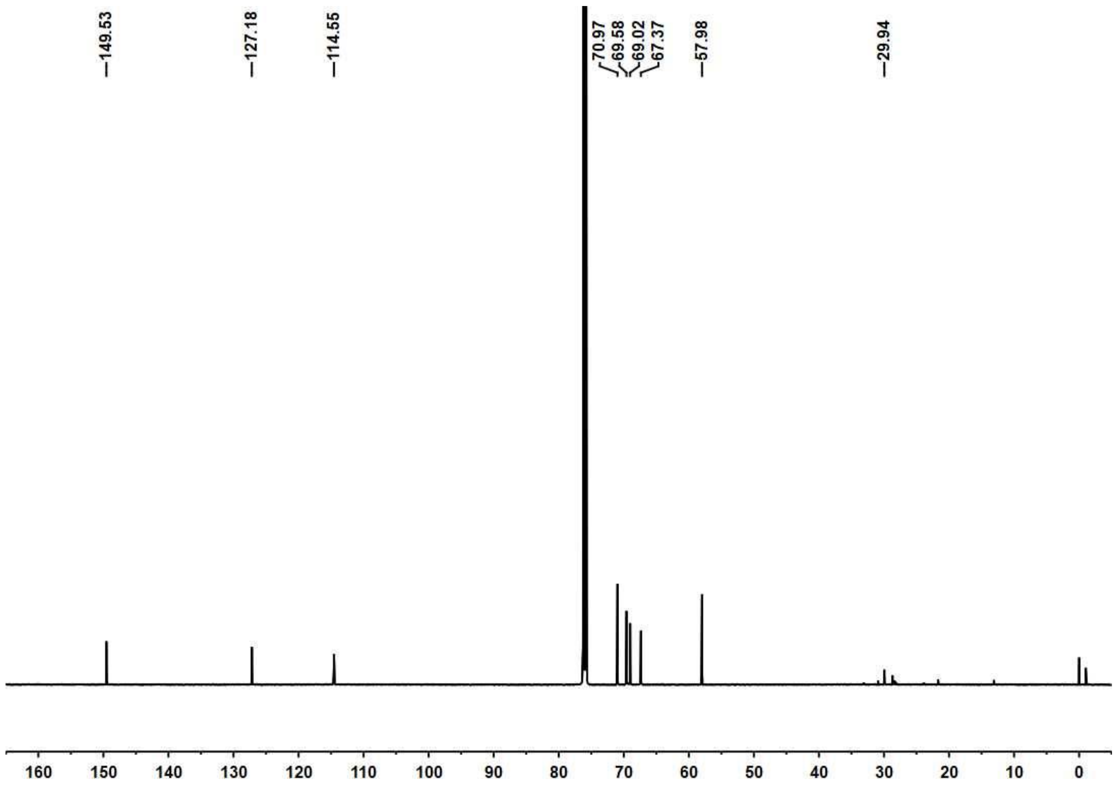

Figure S5. ^{13}C NMR spectrum (150 MHz, $CDCl_3$) of DEP6.

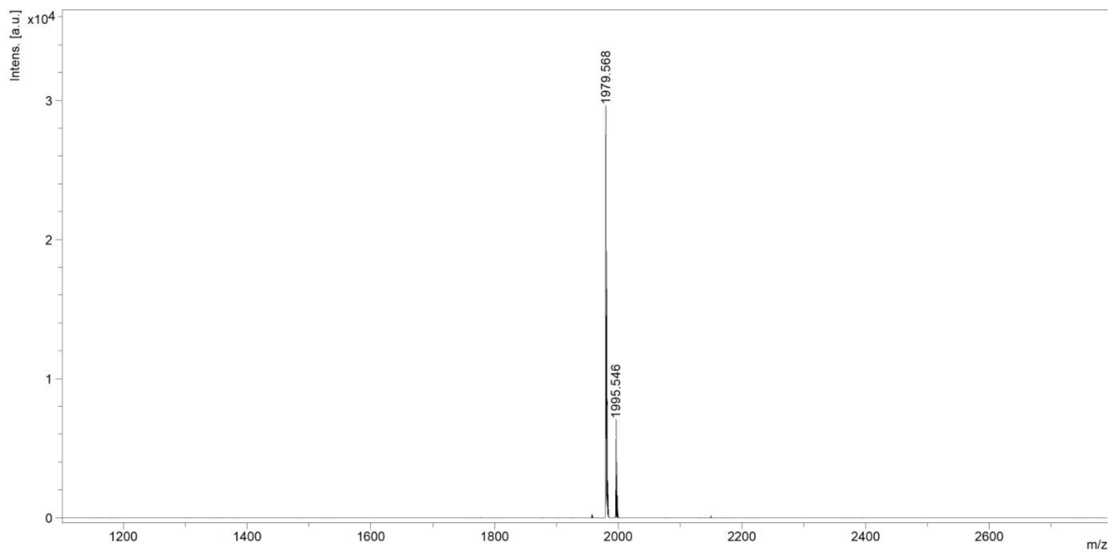

Figure S6. MALDI-TOF-MS of DEP6.

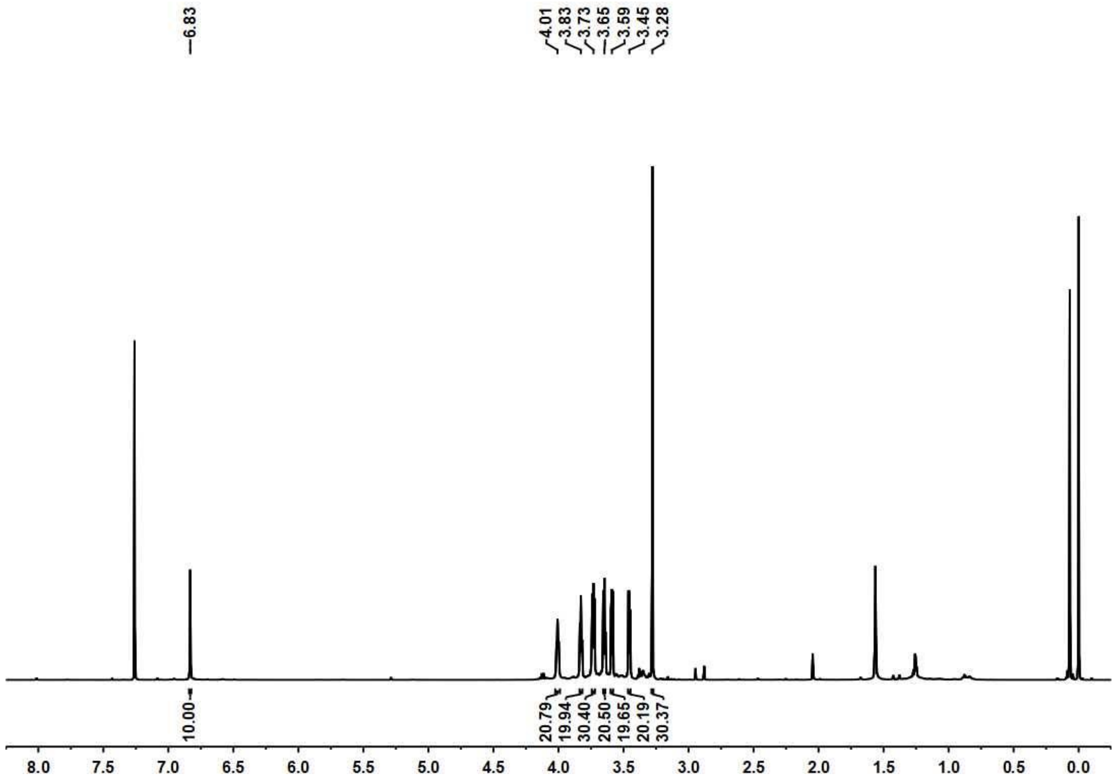

Figure S7. ^{1}H NMR spectrum (600 MHz, $CDCl_3$) of TEP5.

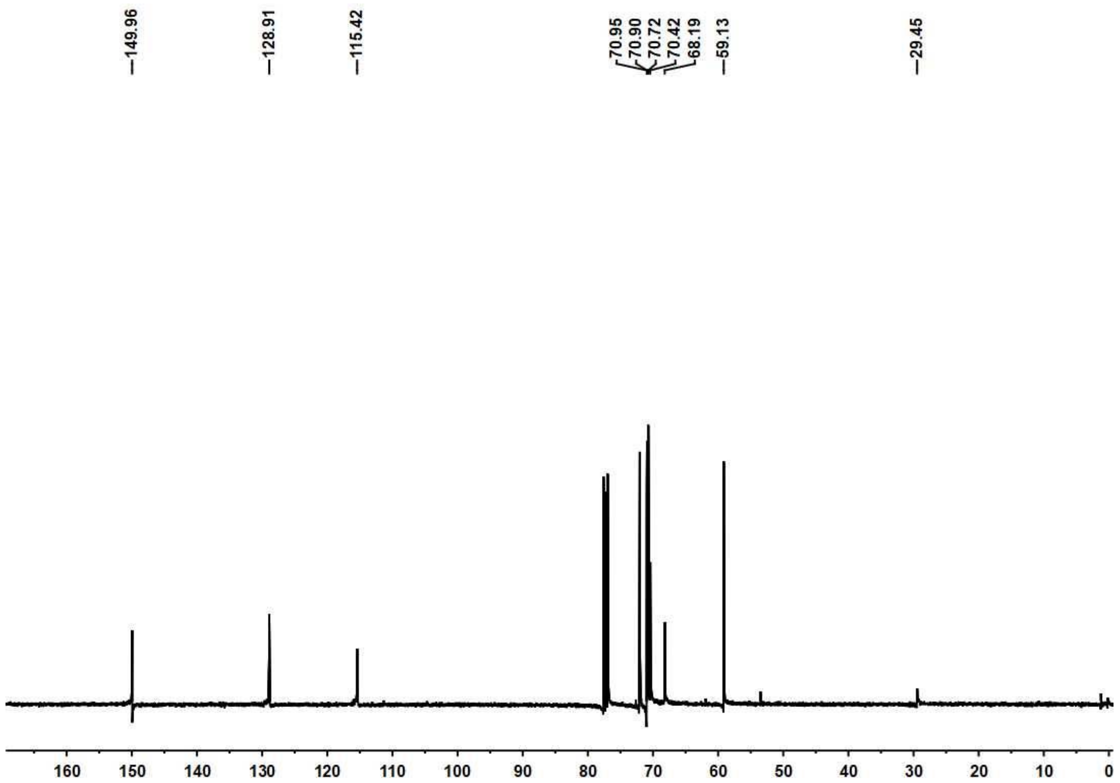

Figure S8. ^{13}C NMR spectrum (150 MHz, $CDCl_3$) of TEP5.

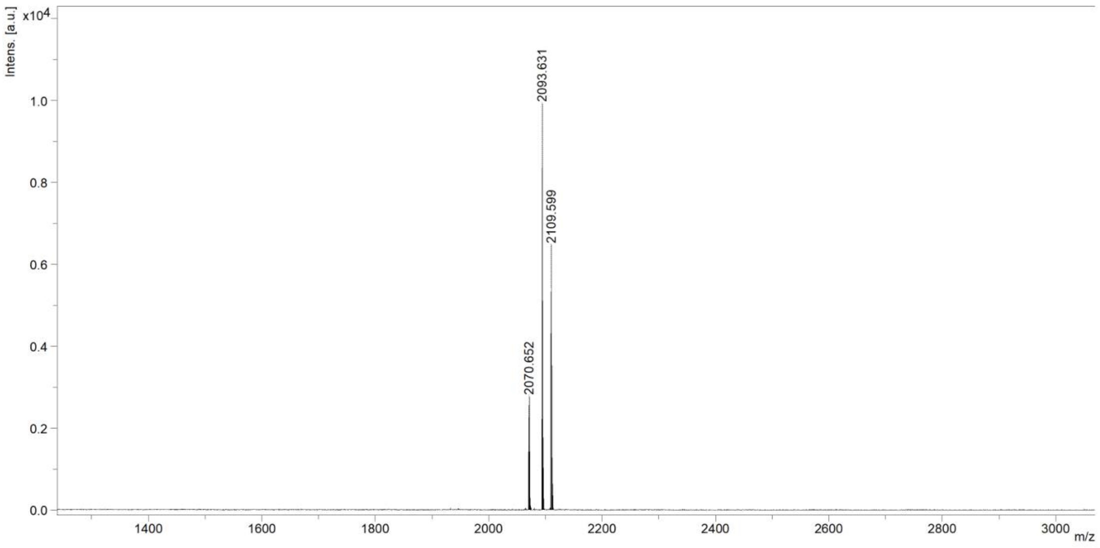

Figure S9. MALDI-TOF-MS of TEP5.

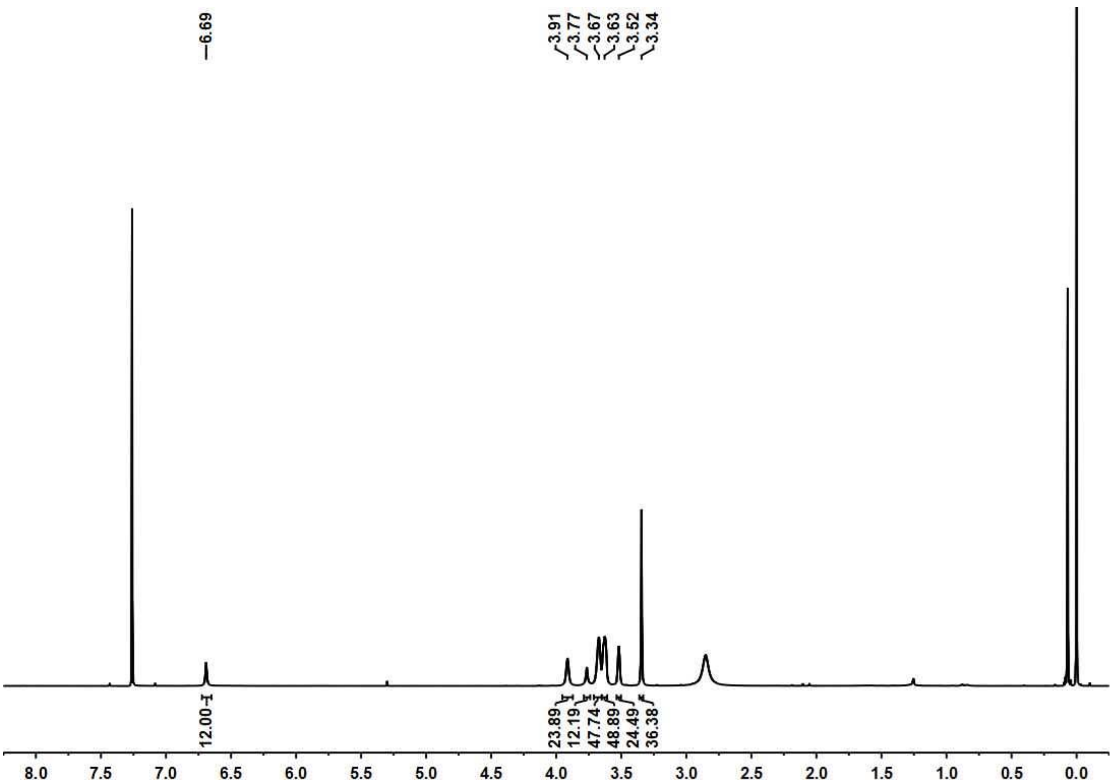

Figure S10. ^{1}H NMR spectrum (600 MHz, $CDCl_3$) of TEP6.

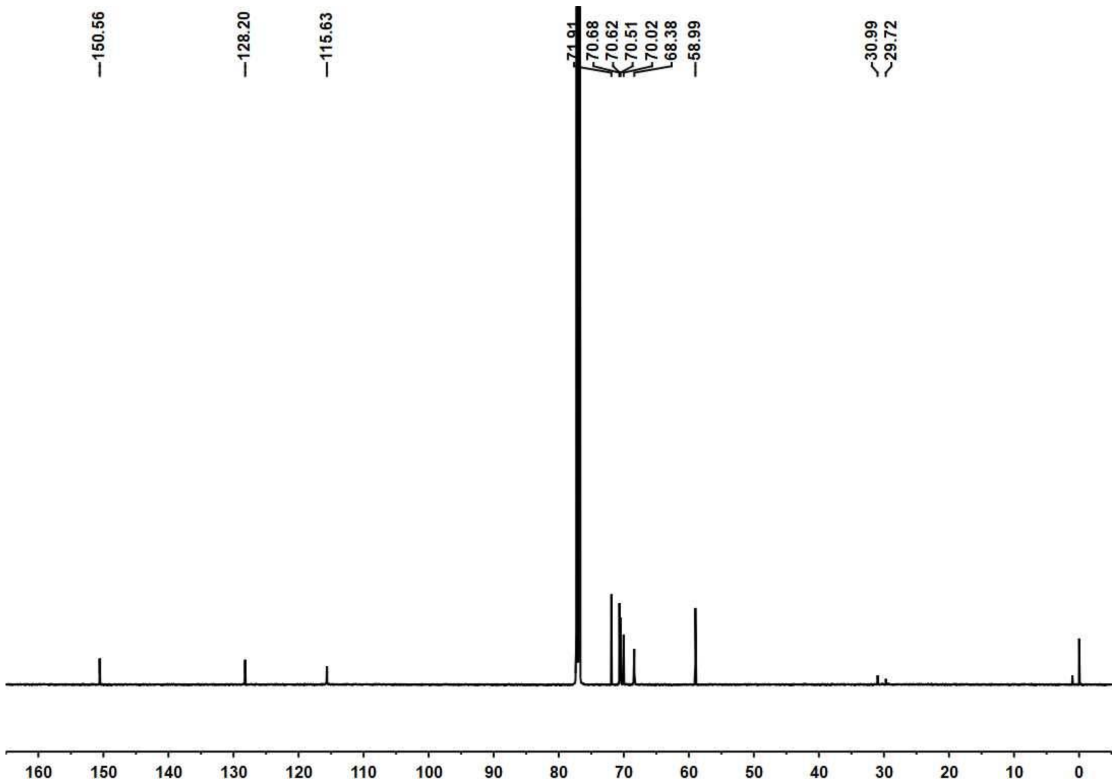

Figure S11. ^{13}C NMR spectrum (150 MHz, $CDCl_3$) of TEP6.

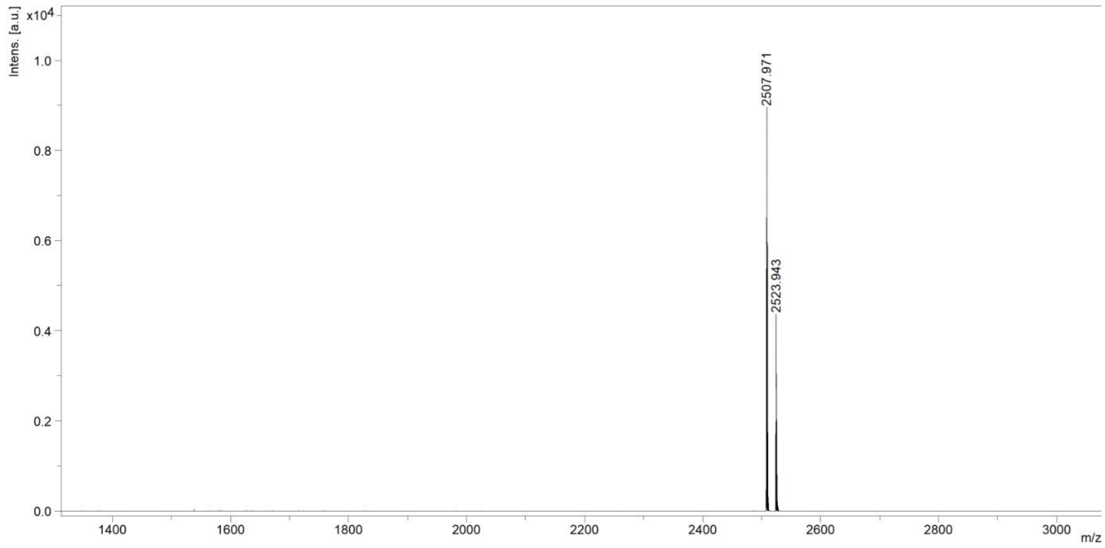

Figure S12. MALDI-TOF-MS of TEP6.

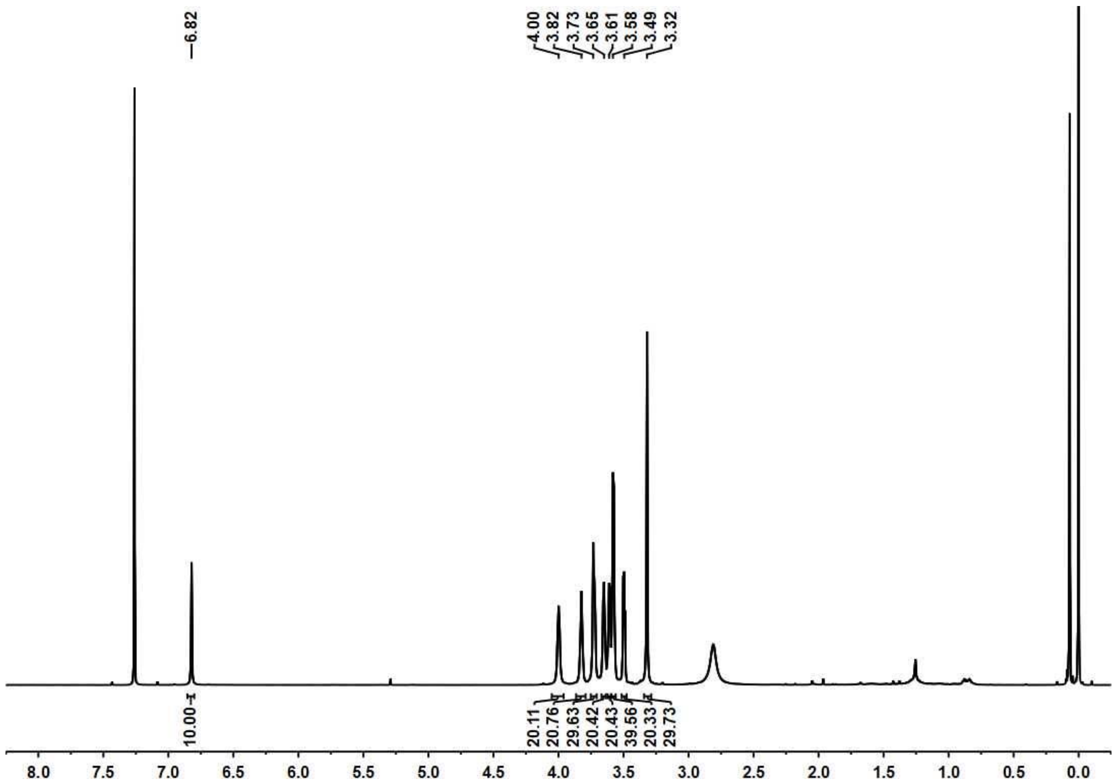

Figure S13. ^{1}H NMR spectrum (600 MHz, $CDCl_3$) of QEP5.

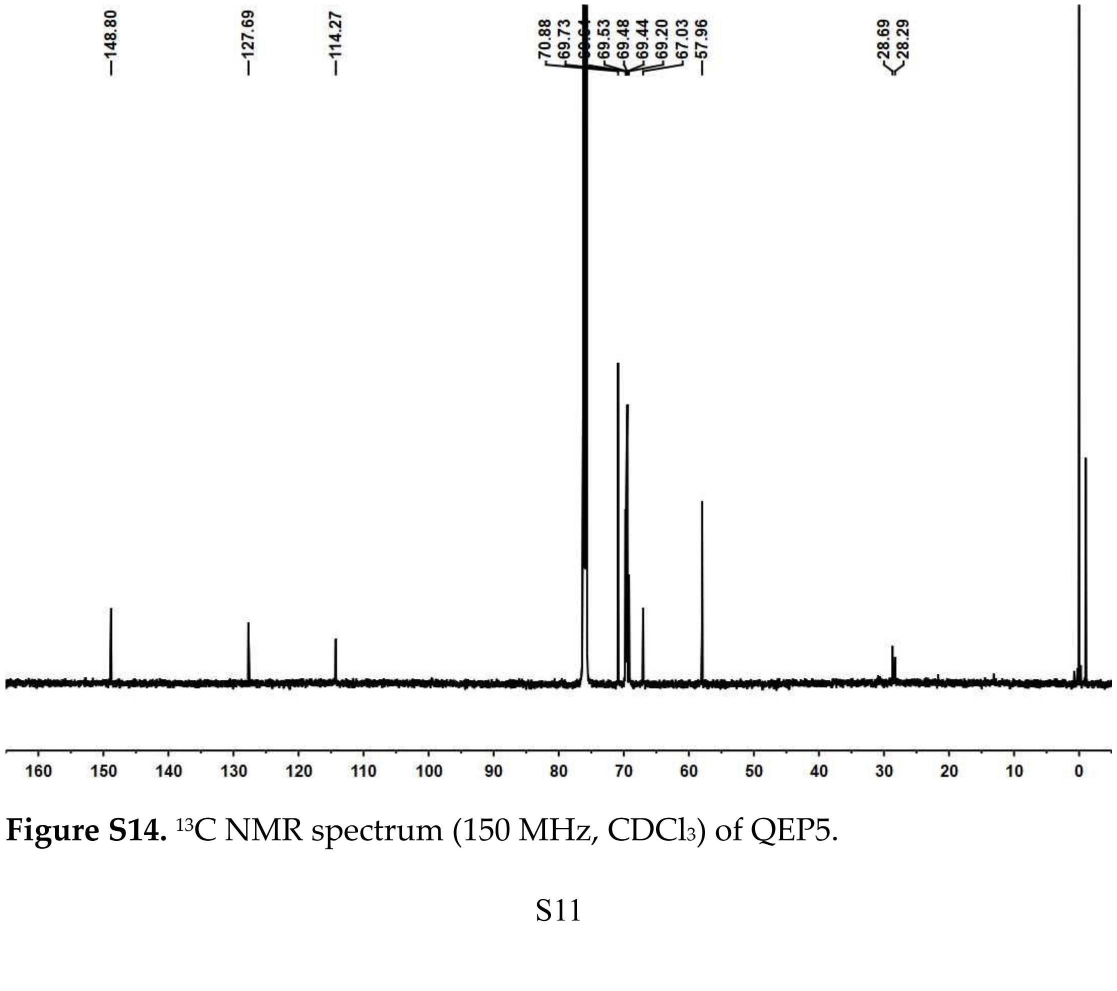

Figure S14. ^{13}C NMR spectrum (150 MHz, $CDCl_3$) of QEP5.

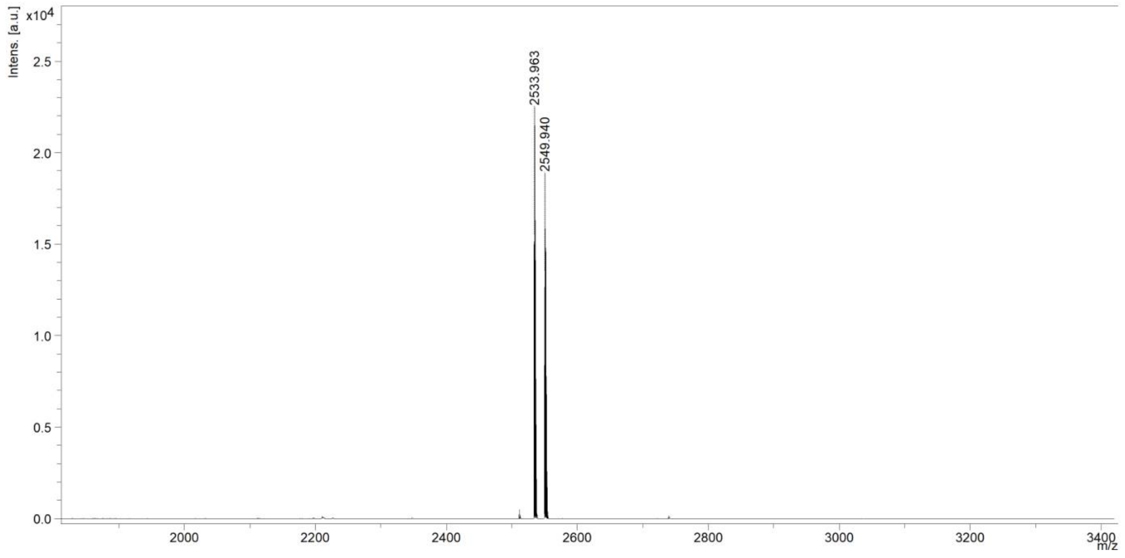

Figure S15. MALDI-TOF-MS of QEP5.

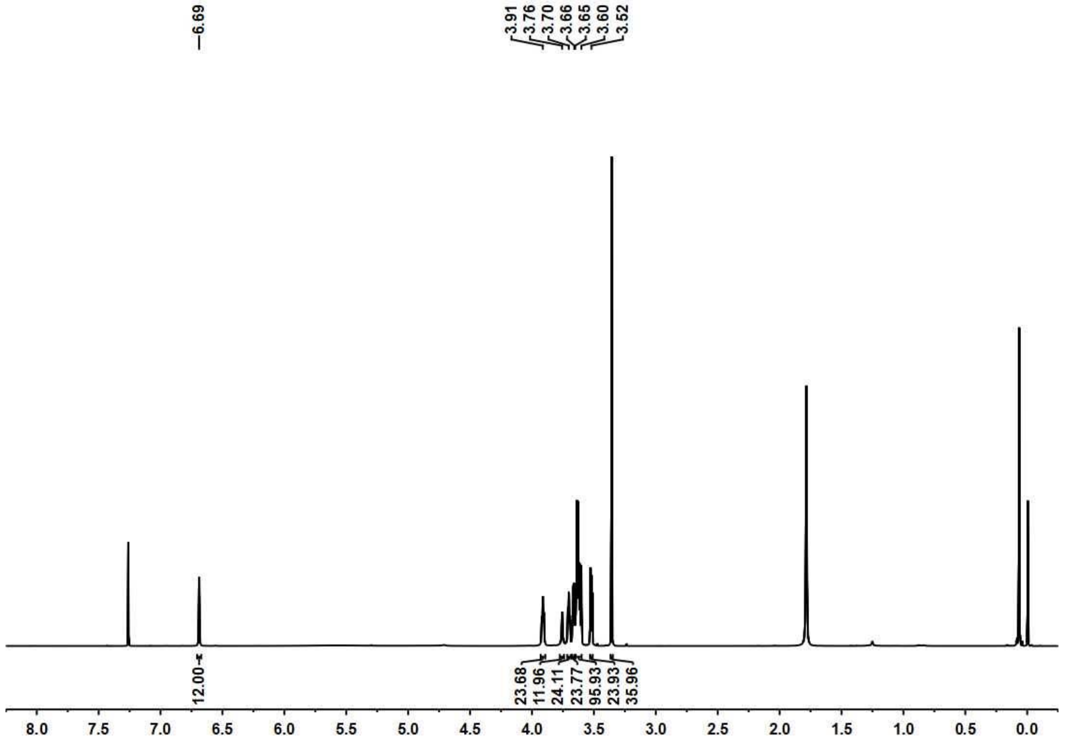

Figure S16. ^{1}H NMR spectrum (600 MHz, $CDCl_3$) of QEP6.

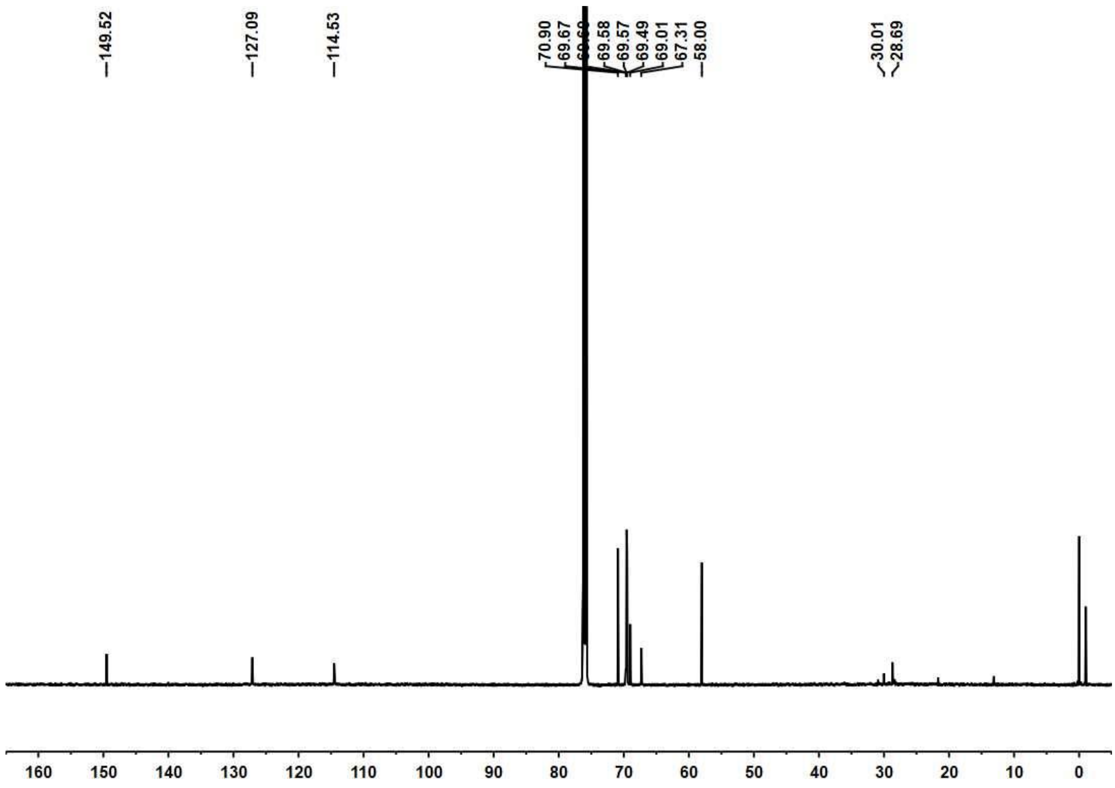

Figure S17. ^{13}C NMR spectrum (150 MHz, $CDCl_3$) of QEP6.

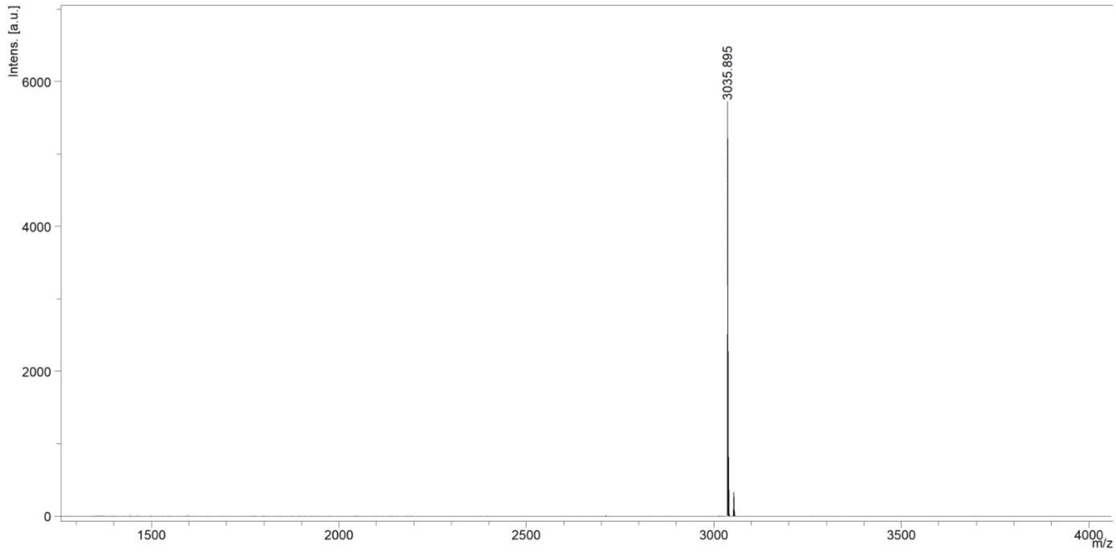

Figure S18. MALDI-TOF-MS of QEP6.

2.2 Viscoelastic spectra for OEPns

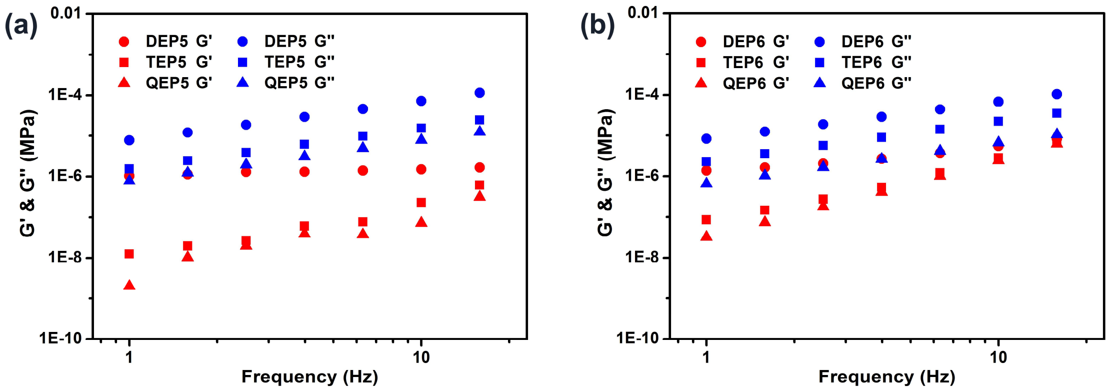

Figure S19. (a) Frequency sweep profile of the storage modulus G′ and loss modulus G′′ of DEP5,TEP5 and QEP5. (b) Frequency sweep profile of storage modulus G′ and loss modulus G′′ of DEP6,TEP6 and QEP6.

2.3 H&E analyses of skin tissue

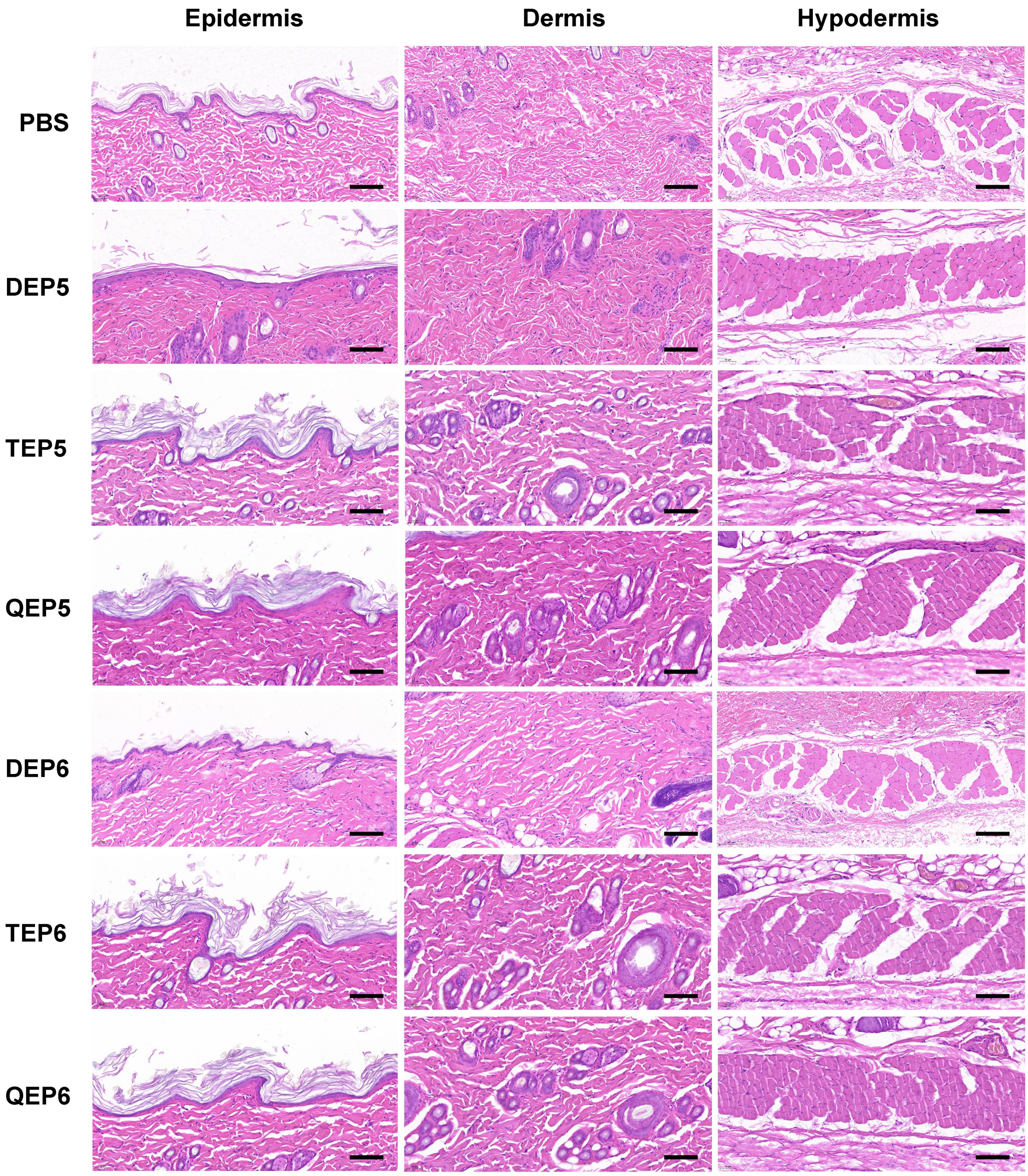

Figure S20. H&E analyses of skin tissue from mice after treatment with (a) PBS, (b) DEP5, (c) TEP5, (d) QEP5, (e) DEP6, (f) TEP6 and (g) QEP6 (scale bar = 100 μm).

2.4 Optimized geometries of ECN with TEP6

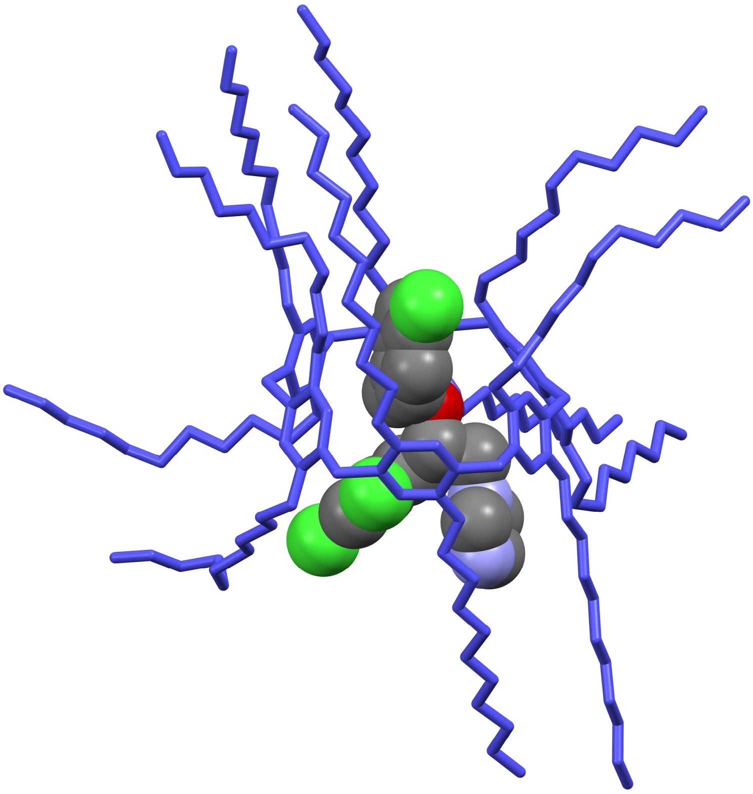

Figure S21. The optimized structure of the MM2 energy-minimized model of the TEP6/ECN complex.

2.5 Calibration curves of ECN

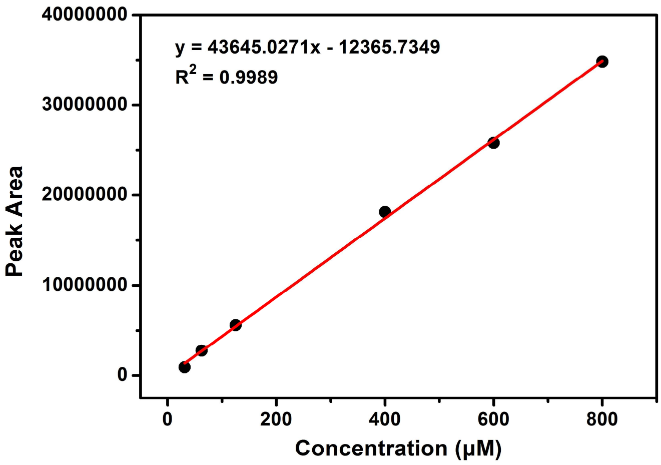

Figure S22. Calibration curve obtained by HPLC and used to calculate the releasing amount of ECN.

3. References

1. Ogoshi, T.; Kitajima, K.; Aoki, T.; Fujinami, S.; Yamagishi, T.-A.; Nakamoto, Y. Synthesis and conformational characteristics of alkyl-substituted pillar[5]arenes. *J. Org. Chem.* **2010**, *75*, 3268–3273.
